# Evaluation of Total Female and Male *Aedes aegypti* Proteomes Reveals Significant Predictive Protein–Protein Interactions, Functional Ontologies, and Differentially Abundant Proteins

**DOI:** 10.3390/insects12080752

**Published:** 2021-08-20

**Authors:** Abubakar Shettima, Shaleni Joseph, Intan H. Ishak, Syahirah Hanisah Abdul Raiz, Hadura Abu Hasan, Nurulhasanah Othman

**Affiliations:** 1 Institute for Research in Molecular Medicine (INFORMM), Universiti Sains Malaysia, Gelugor 11800, Penang, Malaysia; abshetimma400@yahoo.com (A.S.); jshaleni@ymail.com (S.J.); syahirahraiz@usm.my (S.H.A.R.); 2 Department of Microbiology, Faculty of Science, University of Maiduguri, Maiduguri PMB 1069, Nigeria; 3 School of Biological Sciences, Universiti Sains Malaysia, Gelugor 11800, Penang, Malaysia; intanishak@usm.my (I.H.I.); hadura@usm.my (H.A.H.); 4 Vector Control Research Unit, Universiti Sains Malaysia, Gelugor 11800, Penang, Malaysia

**Keywords:** *Ae. aegypti*, female, male, protein identification, LFQ, protein–protein interactions, functional ontologies

## Abstract

**Simple Summary:**

*Aedes aegypti* is a significant vector for flavivirus diseases. Only the female mosquito transmits pathogens, while the male plays a vital role in mating and species continuity. In this study, female and male *Ae. aegypti* proteins were analysed using a mass analyser. Then, we identified proteins for the examination of protein-protein interactions, functional enrichment, and differential protein abundance analysis. This study identified 422 and 682 proteins exclusive to male and female *Ae. aegypti*, respectively, with 608 proteins found in both sexes. The most significant protein-protein interaction clusters and functional enrichments were observed in the biological process, molecular function, and cellular component for the proteins of both sexes. The abundance of the proteins differed, with one protein showing an increase (elongation factor 1 α, EF1α) and two showing reductions (actin family) in females versus males. The study highlights the protein differences in male and female *Ae. aegypti,* and future research could further investigate their roles in mosquito–viral interactions for blocking disease transmission.

**Abstract:**

*Aedes aegypti* is a significant vector for many tropical and subtropical flavivirus diseases. Only the female mosquito transmits pathogens, while the male plays a vital role in mating and species continuity. This study explored the total proteomes of females and males based on the physiological and genetic differences of female and male mosquitoes. Protein extracts from mosquitoes were analysed using LC–ESI–MS/MS for protein identification, protein interaction network analysis, functional ontology enrichment, and differential protein abundance analyses. Protein identification revealed 422 and 682 proteins exclusive to males and females, respectively, with 608 common proteins found in both sexes. The most significant PPIs (<1.0 × 10^−16^) were for common proteins, followed by proteins exclusive to females (<1.0 × 10^−16^) and males (1.58 × 10^−12^). Significant functional enrichments were observed in the biological process, molecular function, and cellular component for the male and female proteins. The abundance of the proteins differed, with one protein showing an increase (elongation factor 1 α, EF1α) and two showing reductions (actin family) in females versus males. Overall, the study verified the total proteomes differences between male and female *Ae. aegypti* based on protein identification and interactions, functional ontologies, and differentially abundant proteins. Some of the identified proteins merit further investigation to elucidate their roles in blocking viral transmission.

## 1. Introduction

*Aedes aegypti* is a primary vector for various pathogens. It is a competent vector for dengue, chikungunya, yellow fever, Zika, and other viruses [1]. The transmission of such arboviruses results in high morbidity and mortality in the tropics and subtropics worldwide. Dengue remains the most significant mosquito-borne arbovirus disease, infecting more than 200 million people annually [2]. Approximately 2.5 billion individuals live in areas at risk for the possible transmission of one or more arboviruses [3]. The global distribution of *Ae. aegypti* is associated with climatic conditions. The optimal temperature of 22 °C to 32 °C is a prerequisite for mosquito development, longevity, and productiveness [4]. Temperatures lower than 10 °C limit larval development and adult survival [5,6,7].

The female *Ae. aegypti* is generally widely studied because it significantly contributes to the transmission of several arboviruses through its exclusive blood feeding. The salivary glands of female *Ae. aegypti* contain proteins that work against the haemostasis of the host blood, which is vital for the mosquito to adapt to feeding on blood and for viral sustenance before transmission [6]. The female *Ae. aegypti* is larger than the male in terms of body size. The females have small palps tinted with silver or white scales and a lyre on their thorax. Male and female *Ae. aegypti* have a pair of plumose antennae, with much denser hair on the antennae in the males than in the females [1,8,9].

The molecular and biological properties of proteins can be easily determined using quick and sensitive proteomic approaches, contributing to the advancement of functional protein catalogues [10]. Mass spectrometry (MS) has enabled the identification of the proteome maps of medically important mosquito species [11,12,13]. Studies on their salivary glands have provided novel insights into the functional characteristics of the various proteins involved in flaviviral transmission [11,12,13]. For instance, MS allows the comprehensive characterisation and understanding of how the adult *Ae. aegypti* midgut peritrophic matrix proteome plays a protective role in the mosquito midgut [2]. The comparative proteome analysis of blood-fed versus sugar-fed female *Ae. aegypti* and a pyrethroid-susceptible strain versus pyrethroid-resistant strain using LC–MS/MS revealed differential protein abundance, including for proteins involved in replication, transcription, and translation driving protein synthesis [14,15]. Proteomics also identified notable proteins differentiated during Zika infections when analysing Wolbachia/Zika coinfection in *Ae*. *aegypti* heads and salivary glands [16]. Moreover, proteomics has deciphered some of the possible mechanisms involved in the occurrence of insecticide resistance in mosquitoes, leading to the potential discovery of biomarkers for detecting resistance in field mosquito populations [17,18,19,20,21]. Bioinformatics tools with which to analyse protein expression from MS data are essential for identifying proteins involved in phenotypic changes [22,23].

In the present study, LC–ESI–MS/MS, known as tandem mass spectrometry (MS), was utilised for protein identification and quantification. First, protein identification was performed to analyse the presence and absence of proteins in male and female *Ae. aegypti*. Furthermore, this study used the label-free quantitative proteomics (LFQ) approach to quantify differentially abundant female versus male proteins. We utilised the electrospray ionisation (ESI) system, coupled to a quadrupole time-of-flight (Q-TOF) analyser for a parallel accumulation of a serial fragmentation acquisition. This model of tandem MS enhanced the speed and sensitivity by increasing the detection of overall mass intensity and resolution for better proteome coverage and quantification [24].

To date, there has been no in-depth analysis of the total proteomes of male and female *Ae. aegypti*. Previous proteomics reports regarding this mosquito species focused on its subproteomes, including those for the midgut [2,25], salivary gland [6,26,27,28], accessory gland, and seminal fluid [29,30]. Hence, this study aimed to supplement the available *Ae. aegypti* proteomic data, covering both mosquito sexes.

Furthermore, this study revealed some significant protein–protein interactions and functional ontologies in female and male *Ae. aegypti*. We also postulated an increased or decreased abundance of proteins in female versus male *Ae. aegypti* by analysing the proteins present in both sexes. This information provides better insight into proteins that researchers could manipulate to block viral transmission in future studies.

Therefore, based on the physiological and genetic differences between female and male *Ae. aegypti*, this study explored the total proteomes of female and male *Ae. aegypti* by protein identification and the determining differentially abundant proteins via LC–ESI–MS/MS.

## 2. Materials and Methods

### 2.1. Mosquito Rearing

*Ae. aegypti* egg papers were obtained from the World Health Organisation (WHO) facility at the Vector Control Research Unit (VCRU) of the Universiti Sains Malaysia (USM) used in this research. The mosquitoes were reared under the optimum conditions of approximately 28 °C and 75% relative humidity at the Insectary of the School of Biological Sciences, Universiti Sains Malaysia. An egg paper with about 300 eggs was placed in an enamel tray with dechlorinated water. After a 24 h incubation, 100 first-instar larvae were transferred to a 3 L enamel tray containing 2 L of water. We fed the larvae food made from dog biscuit powder, beef liver, powdered milk, and yeast in a 2:1:1:1 ratio. The cleanliness of the water was maintained by changing the water every day, and the larval food was constantly replenished until the larvae developed into the fourth instars. Pupae developed from the larvae were transferred using a transfer pipette into 250 mL of water in a disposable plastic cup and placed into a rearing cage to produce adult mosquitoes. Cotton balls were soaked in a 10% sucrose solution and placed in the rearing cage to feed the adult mosquitoes. We used a WHO standard mouth aspirator to select three-day-old female and male adult mosquitoes based on their morphological appearances. The female *Ae. aegypti* is larger than the male in terms of body size, with sparse, tiny hairs on the plumose antennae. The male *Ae. aegypti* has a pair of plumose antennae with hair much denser than that of the female, which is its distinct feature [4,31,32]. Live 3–5-day-old mosquitoes were harvested and kept at −20 °C for 1 min for immobilisation [33]. They were placed in a 2 mL tube on ice and processed for protein extraction at the Institute for Research in Molecular Medicine (INFORMM), USM.

### 2.2. Protein Extraction

A total of 20 adult female and male *Ae. aegypti* mosquitoes were homogenised in 600 µL of CytoBuster^TM^ extraction reagent (Sigma, Darmstadt, Germany) in a mini bead beater using 0.5 mm zirconia beads at 50 rpm for 5 min at room temperature (RT). The pellets were then centrifuged for 5 min at 16,000× *g* at 4 °C. The resultant supernatant was concentrated 6× using 5000 molecular-weight cut-off spin columns (MWCO) (GE Healthcare, Chicago, IL, USA) with centrifugation at 4000× *g* and 4 °C. Finally, a protease inhibitor (Sigma, Darmstadt, Germany) at a 1× final concentration was added to the protein extract, which was then stored at −20 °C. The protein extraction was performed with three biological replicates for the female and male *Ae. aegypti* groups.

The protein concentrations of the mosquito protein extracts were determined using an RCDC^TM^ protein assay kit (Bio-Rad, Hercules, CA, USA) following the manufacturer’s protocol. As a standard, bovine serum albumin (BSA) that was serially diluted 10-fold from a 1 mg/mL stock solution was used. The absorbance at 750 nm was measured using the Spectromic Biomate 3 UV–visible spectrophotometer (Thermo Fisher Scientific, Waltham, MA, USA).

### 2.3. Sodium Dodecyl Sulphate–Polyacrylamide Gel Electrophoresis (SDS–PAGE)

We resolved 20 µg of proteins from each of the three biological replicates of females and males on 10% separating and 3% stacking gels using SDS–PAGE with Biorad Mini Protean Tetra^®^ Systems for electrophoresis (Bio-Rad, Hercules, CA, USA). The electrophoresis system was used to run the proteins at 200 V for 15 min. Then, we stopped the electrophoresis when whole protein complexes were trapped at the top of the separating gel. The gel was stained with RAMA stain made with 0.05% Coomassie Brilliant Blue (CBB), 10% acetic acid, 15% methanol, and 3% ammonium sulphate with gentle rocking for one hour. The staining solution was removed, and the gel was washed with distilled water 3–5 times until single, thick protein bands were visible for each replicate.

### 2.4. In-Gel Protein Digestion

A single, thick protein band for each replicate composed of 20 µg of protein trapped in the gel was cut into small pieces and destained with 200 µL of a destaining solution. The destaining solution was made from 80 mg of ammonium bicarbonate with 20 mL of acetonitrile (ACN) and 20 mL of ultrapure water. The gel pieces were incubated at 37 °C for 30 min with shaking at 300 rpm, and the solution mixture was discarded. We repeated the above step three times to destain the gels completely. The in-gel protein was reduced with 10 mM dithiothreitol (DTT) at 60 °C for 30 min and alkylated with 55 mM iodoacetamide (IAA) for 60 min in the dark at RT. The gel pieces containing the proteins were shrunk with ACN for 15 min at RT. In-gel digestion was performed using 12.5 ng/µL MS-grade trypsin (Promega, Madison, WI, USA) at 37 °C overnight with shaking at 300 rpm. The gels were briefly centrifuged, and the peptides were extracted. To further extract the peptides, 1% trifluoroacetic acid (TFA) and 50% ACN were separately added to the in-gel proteins for 5 min at RT with shaking. Finally, 0.1% TFA in ultra-pure water was added to the in-gel proteins, and the mixture was incubated for 5 min with 300 rpm shaking at RT, which was repeated twice. All of the extracted peptides were pooled and speed-vacuumed until they were dried to the desired volume and subsequently desalted using Zip-Tips (Merck Millipore, Burlington, MA, USA)

### 2.5. LC–ESI–MS/MS

The digested peptides were reconstituted with 30 μL of Buffer A made of 0.1% formic acid in water and centrifuged for 10 min at 150,000 rpm at 4 °C. A 5 µL volume of the digested peptide was loaded and packed into an Agilent large-capacity chip, 300 A, C18 column; 160 nL enrichment column; and 75 µm × 150 mm analytical column (P/N: G4240-62010); buffer A and buffer B composed of 90% ACN in water and 0.1% formic acid, to achieve spatial peptide discrimination. With 20–80% ACN, the gradient pump was eluted for 47 min coupled with a 4 µL/min flow rate using an Agilent 1200 HPLC chip/MS interface connected to an Agilent 6550 iFunnel Q-TOF LC–MS/MS. The method utilised a 47 min gradient starting with a 30 min ramp from 5% to 75% buffer B, followed by a post-run time of 8 min at 75% buffer B. MS was performed using the following parameters: a 1900 V capillary voltage with a 325 °C gas temperature, 5.0 L/min gas flow, auto MS/MS mode, and positive ion polarity setup. Then, data acquisition scanning from 110 to 3000 *m*/*z* for MS and from 50 to 3000 *m*/*z* for MS/MS was conducted. For protein identification, the MS/MS mode spectra were then processed with the data acquisition software PEAKS Studio X^+^ (Bioinformatics Solution Inc., Waterloo, ON, Canada). We used MS1 (MS) and MS2 (MS/MS) data obtained from peptide precursor ions and fragmented ions for protein quantitation and identification. Precursor ions reflect peptide ion intensity, which was used for LFQ quantitation.

### 2.6. Protein Identification Using Automated De Novo Sequencing (PEAKS Studio)

Protein identification using automated de novo sequencing was performed with PEAKS Studio X^+^ (Bioinformatics Solution Inc., Waterloo, ON, Canada) against the UniProt released 2020_01 database specifically for mosquito proteins. Carbamidomethylation was set as a fixed modification, a maximum of 2 missed cleavages were allowed, and trypsin was selected as the enzyme used for digestion. We used a false discovery rate (FDR) of <1% and unique peptides ≥1 for filtering out inaccurate proteins [6]. A −10lgP score greater than 20 indicates relatively high confidence in the detected proteins for further analysis. The −10lgP score is a final peptide score also known as the significance score. It is derived from the *p*-value to predict false-positive rates by calculating the ratio between the number of false identifications above a provided threshold score (T) and the total number of false identifications [34].

### 2.7. Prediction of Protein–Protein Interaction (PPI) Network and Functional Ontology Enrichment

The STRING software database version 11.0b, released on 17 October 2020, was used to predict PPI network associations in identified proteins using the protein identification analysis of male and female *Ae. aegypti*. This study selected the network edge by ‘evidence’, where the coloured lines indicated the type of interaction evidence. The sources of active interactions included ‘text mining’, ‘experiment’, ‘databases’, ‘neighbourhood’, ‘gene fusion’, ‘cooccurrence’, ‘coexpression’, and ‘protein homology’. We chose medium confidence of 40% as the minimum required interaction score and a Markov clustering algorithm (MCL) with three inflation parameters applied with 5% FDR stringency. The functional ontology enrichments in the protein network were retrieved using the analysis tool incorporated in the software. STRING uses the protein accession number from the VectorBase database (VeUPathDB) for analysis. However, this accession number is related to the accession number of the UniProt database, referring to the same protein description. Therefore, the proteins have two formats of accession numbers in this study.

### 2.8. Statistical Analysis for LFQ

Statistical analysis was performed using Perseus 1.5.1.6., referring to the study conducted by Tyanova et al. (2016) [35]. The peptide ion intensity data were uploaded with three replicates for male and female groups for statistical analysis. The other non-statistical data, i.e., protein name, accession number, number of peptides, coverage, and functional ontologies, were also uploaded. Then, the peptide ion intensity data were made logarithmic, using the transformation formula log2(x), to be able to better understand the data in the statistical tests and imputation. A histogram was plotted for each intensity column separately to verify whether the data were normally distributed. Then, the sample was grouped according to its replicates and filtered for valid values, and missing values were imputed from the normal distribution. Quality checking was performed to evaluate the similarity of the same and different groups using multi-scatter plots. Pearson correlations were plotted to analyse the correlations between the groups. A two-sample *t*-test was used to identify the interactors using s0 = 0 and FDR = 0.01. In this analysis, the s0:0 and FDR: 0.01 parameters of the volcano plot were used to show the cut-off curve indicating the proteins that were significantly different in abundance for females versus males. Proteins with a *t*-test difference of >1.5-fold changes and q value < 0.05 were considered significant in this study.

## 3. Results

**Figure 1:**
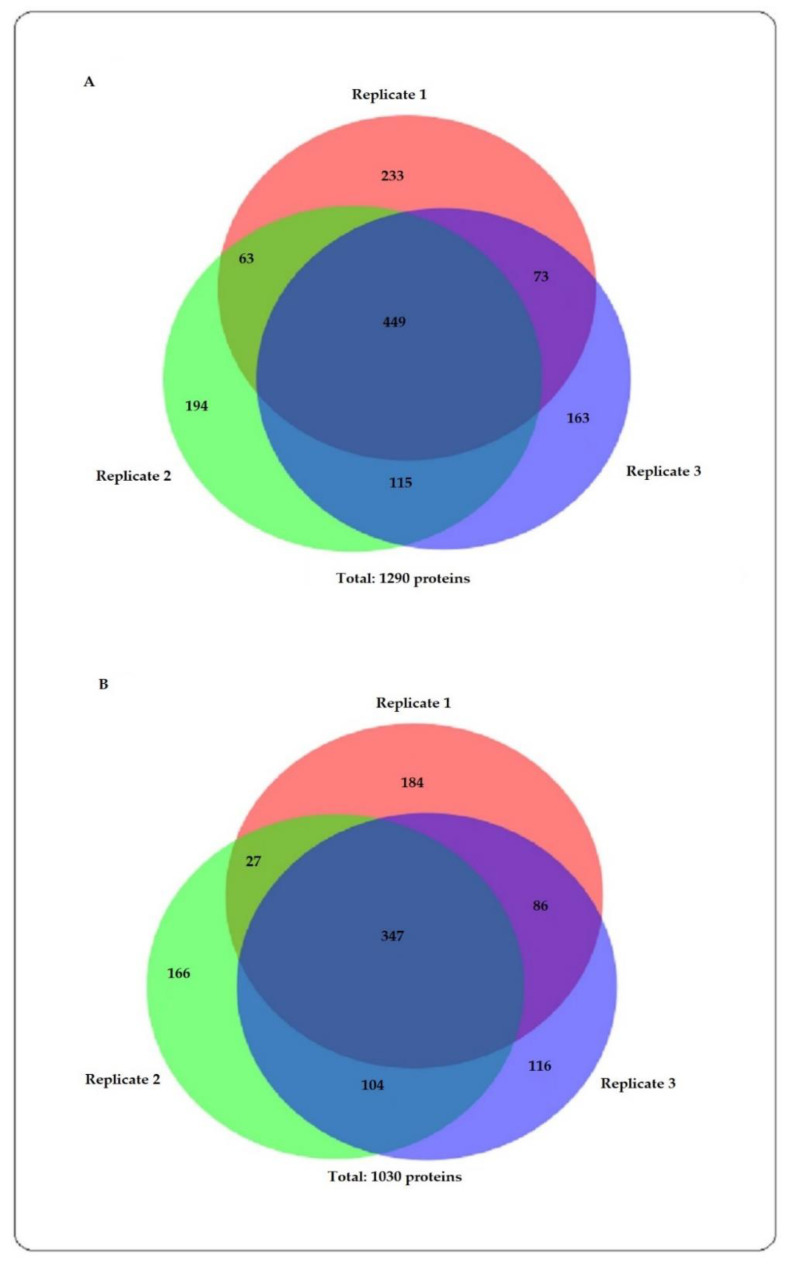
Venn diagrams show protein identification analysis for (**A**) female and (**B**) male *Ae. aegypti*.

Protein identification analysis of all the replicates showed 1030 and 1290 proteins in male and female *Ae. aegypti,* respectively (Figure 1). This analysis also showed that the female *Ae. aegypti* had a higher number of unique proteins, 682, than the male *Ae. aegypti* mosquitoes, with 422.

**Figure 2:**
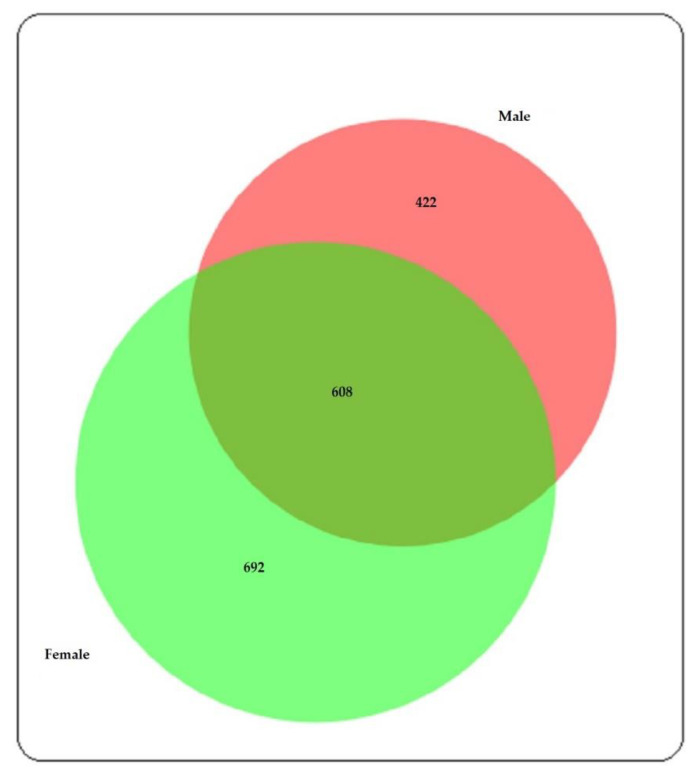
Venn diagram shows proteins commonly and exclusively identified in male and female *Ae. aegypti*.

Meanwhile, the current study identified 608 proteins in both sexes (Figure 2). In addition, the LC–ESI–MS/MS data of this study are available at MassIVE and ProteomeXchange with the identifier numbers MSV000086179 and PXD021571, respectively. The details can be found in Supplementary File S1.

**Figure 3:**
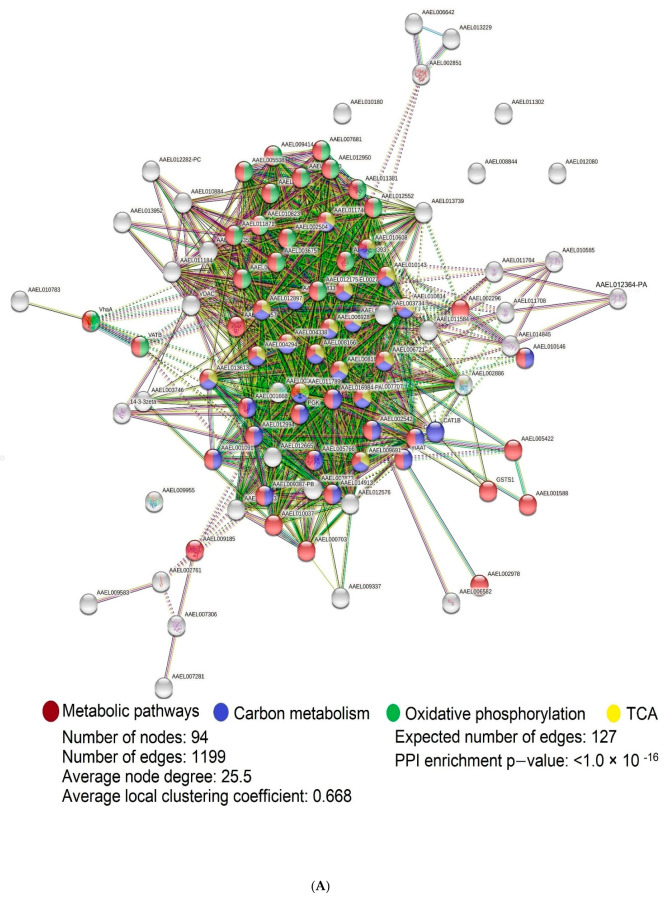

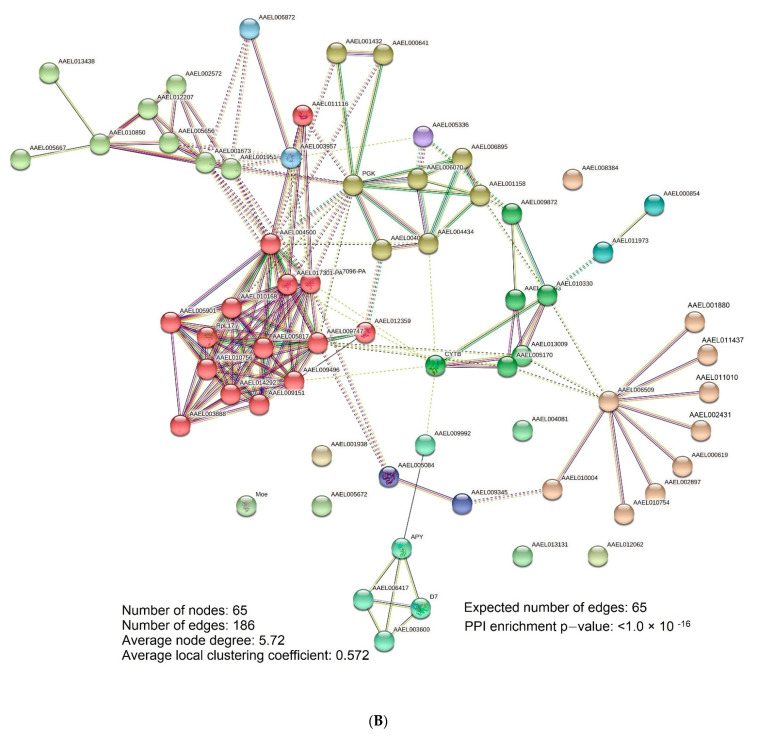

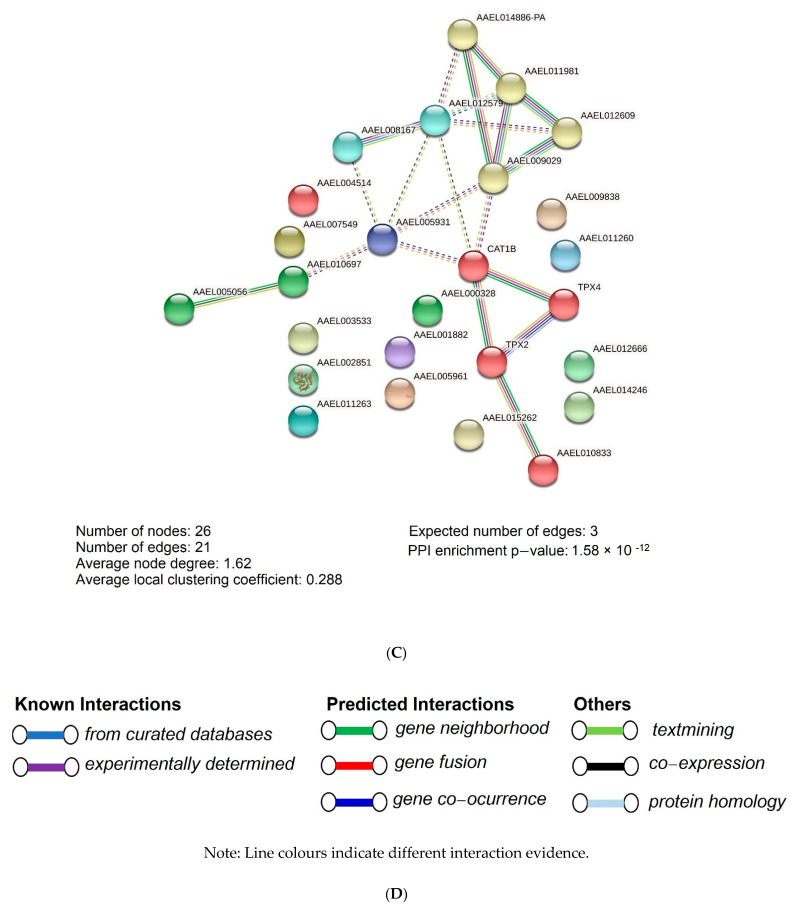
Protein-protein interaction networks using STRING software for proteins identified in *Ae. aegypti*: (**A**) common proteins in females and males; (**B**) proteins exclusive to females; (**C**) proteins exclusive to males. (**D**) Interaction evidence.

STRING analysis showed that the PPI enrichment *p*-value was <1.0 × 10^−16^, suggesting that the network displayed significantly more interactions than expected for the identified proteins in male and female *Ae. aegypti* (Figure 3A). Meanwhile, the analyses indicated more protein–protein interactions than expected for exclusively identified female and male *Ae*. *aegypti* proteins, with PPI enrichment *p*-values < 1 × 10^−16^ and 1.58 × 10^−12^ (Figure 3B,C).

STRING analysis revealed functional ontology enrichments in male and female *Ae. aegypti* proteins, including biological, molecular, and cellular components. The biological process showed six categories: the citrate metabolic process, tricarboxylic acid cycle, antibiotic metabolic process, carbohydrate metabolic process, metabolic drug process, and small-molecule metabolic process (Table 1A). Citrate (Si)-synthase activity and mitochondrial matrix were only observed in the molecular function and cellular component, respectively (Table 1B,C). The significant functional ontology enrichments that were available in the identified proteins exclusive to female *Ae. aegypti* were only observed in the cellular component. The cellular components included ribosomes, the ribonucleoprotein complex, intracellular non-membrane-bounded organelles, the extracellular region, the protein-containing complex, cytoplasm, intracellular organelles, and cells (Table 2). There was no significant functional ontology enrichment observed in the proteins identified exclusively for the male *Ae. aegypti*.

**Figure 4:**
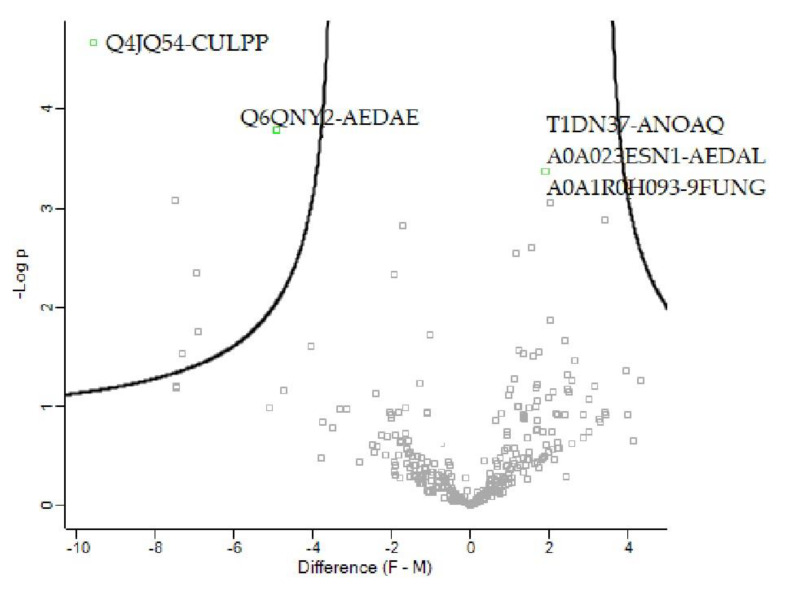
Volcano plot of differentially abundant proteins for female versus male *Ae. aegypti*.

One protein with an increase in abundance and two with a decrease in abundance in female versus male *Ae. aegypti* were identified in this study, as shown in the volcano plot (Figure 4). A0A023ESN1_AEDAL belongs to the elongation factor 1α (EF1α) family proteins (Table 3). The two low-abundance proteins were Q6QNY2_AEDAE and Q4JQ54_CULPP, with actin properties (Table 3).

## 4. Discussion

In this present study, LC–ESI–MS/MS identified more proteins exclusive to female *Ae. aegypti* than to their male counterparts and 682 proteins common for both sexes. From the list of identified proteins, we retrieved 20 and 8 well-annotated/reviewed proteins in females and males, respectively; 140 and 108 putative proteins in females and males, respectively; 117 and 89 uncharacterised proteins in females and males, respectively; 1070 and 824 hypothetical proteins in females and males, respectively. We also noted that there was a limited number of reviewed proteins of *Ae. aegypti* available in the UniProt database and our results. Nevertheless, this study provided early stage validation for identified putative and hypothetical proteins in the database repository, as these data predominantly originated from transcriptomic analyses. The database search using the UniProt_mosquitoes database found significant protein hits belonging to the *Anopheles* species in this study. There were 193 and 311 *Anopheles* proteins matched to unique male and female *Ae. aegypti* proteins, respectively. Furthermore, 361 *Anopheles* proteins were comparable to common proteins for male and female *Ae. aegypti.*

Our previous study showed that the CytoBusterTM extraction reagent (Sigma, Darmstdt, Germany) yielded a higher protein amount, higher number of proteins identified with MS, and higher proteome coverage than did the TCA/acetone precipitation extraction method [36]. Hence, this study used the CytoBuster^TM^ extraction reagent to obtain better proteome coverage for male and female *Ae. aegypti*.

This study is the first to report total protein identification for male and female *Ae. aegypti*, to the best of our knowledge. Although no such research has been conducted, a previous study analysed the specific subproteomes of male and female *Ae. aegypti* [29]. Degner et al. (2019) used tandem MS to profile the male seminal vesicle and female whole ejaculate for the bursae proteins of *Ae. aegypti*. They found that the proteins were transferred from the male to female during the mating process by analysing purified sperm from the male seminal vesicle and the whole ejaculate from the bursae of the mated female. Tandem MS identified 870 sperm proteins and 280 seminal fluid proteins in the female after mating. We compared the lists of male and female proteins in this study to those in the study conducted by Degner et al. (2019) [29] and found 89 male and 112 female proteins present in *Ae. aegypti* that were similar to male sperm/ejaculate proteins. The similarity of the female proteins in our study to sperm/ejaculated proteins is due to the fact that several sperm/ejaculated proteins are found in the seminal fluid of the female *Ae. aegypti* [29]. We found 60 male and 80 female similar proteins in the midgut proteome by comparing the proteins on our protein list to those discussed in the studies conducted by Degner et al. (2019) [29] and Whiten et al. (2018) [2].

Furthermore, 97 similar female and 68 similar male proteins were found in this study and the studies conducted by Whiten et al. (2018) [2]. We compared the list of proteins in this study to the salivary gland proteome and observed four proteins similar to those observed in the female *Ae. aegypti* [37]. The above analysis shows that some proteins can be present in more than one tissue and are not tissue specific. However, many proteins are tissue specific, some of them exclusively found in male and female *Ae. aegypti* based on the above comparison.

We noted that not many proteins in this study were identified in previous studies that were focused on specific tissues. The whole-proteome approach of this study resulted in a different dataset of proteins, compared to that found with the tissue-specific methods. This phenomenon was due to the various protein sources and techniques used. Therefore, the researchers could determine which protein sources and techniques were suitable for the subsequent analysis based on the protein dataset. Overall, this study supplemented the available proteomics data for *Ae. aegypti*.

The STRING network analysis of commonly identified male and female *Ae. aegypti* proteins showed that 56, 31, and 18 proteins were involved in metabolic pathways, carbon metabolism, oxidative phosphorylation, and the tricarboxylic acid cycle, respectively (Figure 3A). The extensive protein interactions in the above pathways suggest versatile roles for these proteins driving cellular respiration and ATP. For example, proteins of the pathways include dihydrolipoyllysine-residue acetyltransferase (AAEL004294), isocitrate dehydrogenase (NAD) subunit, mitochondrial (AAEL000454), pyruvate carboxylase (AAEL009691), and malate dehydrogenase (AAEL007707) (Figure 3A). On the other hand, the most significant cluster, with thirteen mixed proteins exclusive to females, includes mitochondrial F1F0-ATP synthase subunit g/ATP20 (AAEL006509), mitochondrial ATP synthase F chain (AAEL001880), cytochrome b (CYTB), cytochrome c oxidase subunit 4 (AAEL005170), and cytochrome c oxidase subunit 4 (AAEL013009) (Figure 3B). Meanwhile, five proteins exclusive to males involving carbon metabolism and alanine, aspartate, and glutamate metabolism form the most significant network cluster. These proteins are 4-aminobutyrate aminotransferase (AAEL012609), aldehyde dehydrogenase (AAEL009029), aspartate aminotransferase (AAEL012579), aspartate ammonia-lyase (AAEL008167), and 6-phosphogluconate dehydrogenase (AAEL005931) (Figure 3C).

The functional ontology enrichment analysis for common proteins in both sexes showed that the identified proteins were involved in six significant categories of the biological processes (Table 1(A)). Two proteins—probable citrate synthase 1, mitochondrial (AAEL002956), and probable citrate synthase 2, mitochondrial (AAEL011789)—are involved in all the categories, i.e., the carbohydrate metabolic process, tricarboxylic acid cycle, citrate metabolic process, antibiotic metabolic process, carbohydrate metabolic process, drug metabolism, and small-molecule metabolic process. In addition, under the molecular function and cellular compartment, these two proteins are associated with citrate (Si)-synthase activity and the mitochondrial matrix (Table 1B,C). Regarding citrate synthase, Wasinpiyamongkol et al. (2010) reported the upregulation of the citrate synthases 1 and 2 in the salivary glands of sugar-fed versus blood-fed *Ae. aegypti* using 2DIGE MALDI-TOF/MS analysis [38]. The results showed the upregulation of citrate synthases 1 and 2, with a 1.68-fold change in sugar-fed versus blood-fed females in the *Aedes* salivary glands. The researchers suggested that citrate synthases 1 and 2 have housekeeping functions. These enzymes are involved in glycolysis, the tricarboxylic acid cycle, and energy generation from the cell’s mitochondria. They also perform oxidative metabolism, which subsequently drives ATP synthases for overall cellular respiration. Almeras et al. (2010) identified a mitochondrial ATP synthase subunit as part of the salivary protein repertoire of *Ae. aegypti* [28]. It is a highly conserved protein and recognised as a potential marker for screening travellers exposed to mosquitoes in endemic areas. The ubiquitous presence of these two proteins in all the functional ontologies and categories suggests a significant role for them in the energy metabolism and cellular respiration pathways, ensuring optimum physiological functions in the mosquito.

Furthermore, the V-type proton ATPase catalytic subunit A (VhaA) was involved in the drug metabolism and small-molecule metabolic process (Table 1A). VhaA is an essential electrogenic pump with ATPase activity. It can transport protons from the cytoplasm to the extracellular fluid, thereby acidifying organelles to generate negative cell membrane voltages. The membrane voltage drives ion transport through specific ion channels, creating electrochemical proton potential. In addition, the electrochemical proton potential can facilitate secondary active transport, such as cation/H+ exchange or anion/H+ co-transport [27].

The identified proteins are exclusive to female *Ae. aegypti* and revealed significant functional ontology enrichment only in the cellular component (Table 2). In the ribosome component, two identified proteins were 40S ribosomal protein S3a (AAEL005901) and 60S ribosomal protein L17 (RpL17), with functions in the structural constituent of ribosome and RNA translation, respectively (Table 2) [39]. These ribosomal proteins are also present in all the enriched functional categories except for the extracellular region component. The intracellular non-membrane-bounded organelle cytoplasm, intracellular organelle, and cell components expressed the moesin (Moe) protein, with actin-binding properties (Table 2).

Regarding moesin, Sandiford et al. (2015) [40] previously indicated actin secretion in the haemolymph of female *Anopheles gambiae.* They injected the mosquito with bacterial lipopolysaccharide (LPS) to examine the haemocytes. In the study, they added 1.9 × 10^5^ *E. coli* (MOI 1000) bacteria into a supernatant comprising actin secreted by the Moss55 mosquito cell line. The outcome was a 96% reduction in the actin present in the supernatant fraction. The results indicated that *E. coli* had sequestered actin from the mosquito haemolymph by binding to the actin. This bacterium–actin binding resulted in immune system induction and subsequent bacterial elimination in the mosquito midguts through activation of the Toll pathway.

Cytochrome b (CYTB) is part of the mitochondrial respiratory chain, functioning as a respiratory electron transport chain, and possesses oxidoreductase activity. It is involved in protein-containing complexes, the cytoplasm, intracellular organelles, and cell components (Table 2). Zhao et al. (2009) used qPCR to determine the expression levels of cytochrome b in the different developmental stages for mosquitoes, namely, eggs, larvae, pupae, and adults [41]. Low expression of cytochrome b was observed in the late larval and pupal stages, and it was expressed at different levels during the egg and early larval instar stages. In adults, the expression of cytochrome b was higher in females than in males, at all ages. The authors reported that the expression of cytochrome b was unnoticeable in older male mosquitoes compared to teneral and older male and female *Ae. aegypti*. The lack of detectable cytochrome b expression in older males in the study conducted by Zhoa et al. (2009) may explain why the functional ontology enrichment for male *Ae. Aegypti* was not significant in this study.

Apyrase (APY) and D7 protein were only present in the extracellular region components (Table 2). Apyrase promotes hematophagy, prevents platelet aggregation in the host through ADP dependency, and limits the blood probing time. Apyrase also facilitates rapid blood meal acquisition for hematophagous insects [41,42]. Protein D7 is a salivary gland allergen with odorant-binding activity, and it enhances blood feeding in female mosquitoes [43].

Oktarianti et al. (2015) reported that Apyrase and D7 were the major identified immunogenic proteins in the salivary glands of female *Ae. aegypti* using SDS–PAGE and QTOF–LC–MS/MS analysis [44]. These two proteins were among the secreted proteins responsible for the interplay between mosquitoes and their hosts during blood feeding [28]. The functional ontology enrichment of Apyrase and D7 proteins in female *Ae. aegypti* verified that they were significantly involved in the blood-feeding process. Therefore, these proteins could be used as potential markers for screening travellers exposed to this vector in endemic areas.

STRING analysis did not reveal significant functional ontology enrichment in proteins exclusive to males in this current study. This phenomenon may be due to the many unreviewed or putative proteins in the male protein list, and their functional protein domains are not well studied. We also noted fewer reviewed proteins deposited into the STRING database, which hindered the identification of these putative proteins in male *Ae. aegypti*.

This study showed a high abundance of an isoform of EF1α (A0A023ESN1_AEDAL) with a 1.8433-fold change in female versus male *Ae. aegypti* (Table 3). The EF1α protein has several biological functions, including protein synthesis, actin binding, and apoptotic regulation [45]. Several isoforms of EF1α are found in eukaryotic organisms, including arthropods. Previous studies investigated mosquitoes infected with viruses or viral proteins to evaluate EF1α’s interactions with viral proteins, RNA, and replication [44,46,47,48]. For example, Colpitts et al. (2011) identified an interaction between mosquito EF1α and flaviviral proteins, which acted as binding partners, using tandem affinity purification assay (TAP) and LC–MS/MS for peptide sequencing and identification [49]. They determined an increased expression of EF1α in the *Ae. albopictus* C6/36 mosquito cell line upon DENV and West Nile virus infections. They further examined the protein expression level using qPCR after viral infections of the *Ae. albopictus* C6/36 mosquito cell line. DENV and West Nile viral infections increased the expression of EF1α, with 1.9- and 1.6-fold changes, in the *Ae. albopictus* C6/36 mosquito cell line, respectively.

On the other hand, EF1α has also been identified in the midgut of female *Ae. aegypti* infected with DENV-2 and four serotypes in a virus competence study [13]. Muñoz et al. (2013) identified this protein using an affinity chromatography assay, followed by LC–MS/MS for protein identification for the mosquito midgut cells of *Ae. albopictus* C6/36 challenged with DENV. The results suggested an increased expression of the EF1α protein during viral infection and replication, emphasising its roles in binding and translation. Furthermore, EF1α proteins have also been reported in the extracellular vesicles of DENV-infected *Ae. aegypti* [50] and are related to translational apparatus [49].

Based on this study and previous findings, we postulate that reducing the protein expression level of EF1α in female *Ae. aegypti* will reduce the viral binding efficiency and replication process. Therefore, this protein molecule merits further investigation for functional analysis using molecular genetic technology to decrease the expression of EF1α in female *Ae. aegypti*. We suggest the use of the midgut tissue for protein functional analysis. The outcome could help to reduce or block the transmission of mosquito–viral diseases to humans.

The two decreased abundant protein isoforms in the female versus male *Ae. aegypti* are AAEL001951-PA and actin (Q6QNY2_AEDAE and Q4JQ54_CULPP), with 4.9- and 9.5-fold changes, respectively. All phagocytic activities in mosquitoes involve restructuring the actin cytoskeleton [26,51]. Meanwhile, mosquito cytoplasmic actin has many biological functions, including cell signalling and immunity [52].

In the mosquito innate immune system, actin is essential in fighting infections [52]. Therefore, understanding the role of proteins in innate mosquito immunity is vital, as mosquitoes are vectors of several human diseases. Sandiford et al. (2015) switched off/silenced actin using dsRNA in *An. gambiae* before feeding them with a *P. falciparum* gametocyte culture [40]. The researchers found that the gametocyte infection intensity increased 3.4-fold in the midguts of the actin-silenced *An. gambiae*.

Actin in *Ae.* *aegypti* was reported to be the binding partner of DENV-2 [50]. Acosta et al. (2008) prevented actin polymerisation by inhibiting the actin microfilaments of *Ae. albopictus* C6/C3 cell lines using cytochalasin D, significantly reducing the virus titre during virus entry [53]. Zhang et al. (2013) confirmed that actin mRNA was abundant in the salivary gland and *Ae. albopictus* C6/C3 cell lines [50]. The expression of actin was upregulated at eight days after DENV-2 infection but rapidly declined. The decline in actin expression may be related to involvement in DENV-2 entry in the salivary gland.

Our results show a highly decreased abundance (>4-fold-change) of actin proteins in female versus male *Ae. aegypti*. However, actin polymerisation can still facilitate virus entry into *Aedes* mosquitoes with a lower abundance of actin. Therefore, our findings are in agreement with those of a previous study suggesting that inhibiting actin depolymerises this protein and prevents virus entry. Finally, this may reduce or block mosquito–human viral transmission.

## 5. Conclusions

Overall, this study supplemented the proteomic data for male and female *Ae. aegypti* and highlighted the proteome difference between the sexes. In addition, it provided better insight into the mosquito’s biological system. The differential abundance of proteins merits further investigation, mainly for combating mosquito–viral disease transmission.

## Figures and Tables

**Table 1:** Functional ontology enrichment of proteins identified in male and female *Ae. aegypti*.

**(A) Biological Process**						
**Term ID**	**Term** **Description**	**Observed** **Gene Count**	**Background** **Gene Count**	**Strength**	**False** **Discovery Rate**	**Matching** **Proteins** **in the** **Network (Labels** **)**
GO:0005975	Carbohydrate metabolic process	2	5	1.83	0.0126	AAEL002956AAEL011789
GO:0006099	Tricarboxylic acid cycle	2	2	2.23	0.0126	AAEL002956 AAEL011789
GO:0006101	Citrate metabolic process	2	2	2.23	0.0126	AAEL002956AAEL011789
GO:0016999	Antibiotic metabolic process	2	3	2.05	0.0126	AAEL002956AAEL011789
GO:0017144	Drug metabolic process	3	21	1.38	0.0126	AAEL002956 VhaA AAEL011789
GO:0044281	Small-molecule metabolic process	3	31	1.21	0.0126	AAEL002956 VhaA AAEL011789
**(B) Molecular Function**						
GO:0004108	Citrate (Si)-synthase activity	2	2	2.23	0.0101	AAEL002956 AAEL011789
**(C) Cellular Component**						
GO:0005759	Mitochondrial matrix	2	4	1.92	0.0097	AAEL002956 AAEL011789

**Table 2:** Functional ontology enrichment of cellular component proteins identified exclusively in female *Ae. aegypti*.

Term ID	Term Description	Observed Gene Count	Background Gene Count	Strength	False Discovery Rate	Matching Proteins in the Network (Labels)
GO:0005737	Cytoplasm	4	119	0.91	0.0211	AAEL005901 Moe RpL17 CYTB
GO:0005840	Ribosome	2	14	1.54	0.0211	AAEL005901 RpL17
GO:0032991	Protein-containing complex	3	77	0.98	0.0211	AAEL005901 RpL17 CYTB
GO:0043229	Intracellular organelle	4	135	0.86	0.0211	AAEL005901 Moe RpL17 CYTB
GO:0043232	Intracellular non-membrane-bounded organelle	3	36	1.31	0.0211	AAEL005901 Moe RpL17
GO:1990904	Ribonucleoprotein complex	2	16	1.48	0.0211	AAEL005901 RpL17
GO:0005576	Extracellular region	2	24	1.31	0.0219	APY D7
GO:0005623	Cell	4	186	0.72	0.0271	AAEL005901 Moe RpL17 CYTB

**Table 3:** Proteins with increased and decreased abundance in female versus male *Ae. aegypti*.

Name	Accession Number	Fold Change (*t*-Test Difference)	q-Value	Abundance
Elongation factor 1α (fragment)	A0A023ESN1_AEDAL	1.8433	0.017	Increased
AAEL001951-PA	Q6QNY2_AEDAE	4.91199	0.034	Decreased
ACTIN	Q4JQ54_CULPP	9.55168	0.00	Decreased

## Data Availability

LC–ESI–MS/MS data of this study is available at MassiVE and ProteomeXchange with identifier numbers MSV000086179 and PXD021571, respectively.

## Supplementary Materials

The following are available online at https://www.mdpi.com/article/10.3390/insects12080752/s1, Supplementary File S1. (List of proteins identified in this study).
